# Association Between Executive Dysfunction and Relapse of Alcohol Dependence After Deaddiction Treatment – a Cross-Sectional Study From India

**DOI:** 10.1192/bjo.2022.248

**Published:** 2022-06-20

**Authors:** Pavithra Ethirajan, Manjula Simiyon, Manikandan Mani, Jiann Loo, Pradeep Thilakan

**Affiliations:** 1 Pondicherry Institute of Medical Sciences, Pondicherry, India; 2 Betsi Cadwaladr University Health Board, Wrexham, United Kingdom

## Abstract

**Aims:**

To assess the association between executive dysfunction and the severity of alcohol dependence, age at first drink, and the number of deaddiction treatments in the past (indicating current relapse).

**Methods:**

This cross-sectional study was carried out in the in-patient unit of the Department of Psychiatry of a tertiary care teaching hospital in South India. Institutional Ethics committee approval was obtained. 43 Adult patients who were diagnosed with alcohol dependence syndrome according to the International Classification of Diseases (ICD-10), admitted for deaddiction treatment, whose withdrawal symptoms were adequately treated, and who did not have a severe mental or physical illness were included in the study. The hospital has the policy to offer 21 days of inpatient treatment which comprises of detoxification, motivation enhancement therapy, group therapy, and family interventions. Only those patients previously treated in the same hospital were considered to maintain, homogeneity. After briefing the patients about the procedure, a participant information sheet was provided and informed consent was obtained. A semi-structured proforma was used to collect the demographic details and information regarding alcohol consumption. AUDIT (Alcohol Use Disorder Identification Test) Questionnaire was used to assess the severity of alcohol dependence. After familiarising the participants with the study and the procedure, their executive function was assessed using Frontal Assessment Battery (FAB).

**Results:**

The mean age of the participants was 36(SD 7.8) years. The mean age of the first drink was 21 years (SD 6). The mean duration of alcohol dependence was 5.6(SD 3.6) years. The total number of de-addiction treatments was 2.2(SD 1.2). 58% at least had a middle school education, 30% were unemployed and 48.2% belonged to lower socioeconomic status. 72% were married and 60% had a family history of significant alcohol consumption. The average duration of total abstinence was 147.5(SD 74) days. 86% consumed spirits and 14% consumed arrack regularly. The mean score on the AUDIT scale was 26.8 (SD 7.3). The mean FAB score was 11.2(SD 4.2). 53.4% had a score of less than or equal to 12 indicating executive dysfunction. Age, (p-value-0.02) number of de-addiction treatments (p-value- 0.014) and the AUDIT score (p-value-0.04) had statistically significant negative correlation with the FAB score. Age had a positive correlation with the number of de-addiction treatments

**Conclusion:**

There is a bidirectional relationship between alcohol use and executive dysfunction. By establishing a significant association between executive dysfunction and the number of de-addiction treatments indicating relapses, this study reiterates the importance of assessing executive dysfunction among this population, to prevent relapses. It can be used as a high-risk indicator for relapse and adequate preventive measures should be in place while treating these patients.

